# Signature of circular RNAs in peripheral blood mononuclear cells from patients with active tuberculosis

**DOI:** 10.1111/jcmm.14093

**Published:** 2018-12-18

**Authors:** Yurong Fu, Jindong Wang, Jinjuan Qiao, Zhengjun Yi

**Affiliations:** ^1^ Department of Medical Microbiology of Clinical Medicine College Weifang Medical University Weifang Shandong Province China; ^2^ Department of Laboratory Medicine, Key Laboratory of Clinical Laboratory Diagnostics in Universities of Shandong Weifang Medical University Weifang Shandong Province China

**Keywords:** active tuberculosis, biomarkers, circRNAs, peripheral blood mononuclear cells, signalling pathway

## Abstract

The study was to characterize the expression profiles of circular RNAs (circRNAs) in peripheral blood mononuclear cells (PBMCs) from active tuberculosis (TB) patients and to investigate their function. Microarray was applied to detect circRNA expression and reverse transcription‐quantitative polymerase chain reaction was conducted to validate the microarray results. Meanwhile, receiver operating characteristic curve (ROC) curve was calculated to evaluate the predictive power of the selected circRNAs for TB diagnosis. Additionally, circRNA/miRNA interaction was predicted based on miRNA target prediction software, and gene ontology as well as Kyoto Encyclopedia of Genes and Genomes pathway analysis were used to predict their biological function. In total, 171 circRNAs were found to be dysregulated in TB samples**. **Specifically, circRNA_103017, circRNA_059914 and circRNA_101128 were confirmed to be increased, while circRNA_062400 was decreased in TB samples. ROC analysis revealed that circRNA_103017 had potential value for TB diagnosis, followed by circRNA_059914 and circRNA_101128. Moreover, circRNA_101128 expression in TB samples was negatively correlated with the level of its possible target let‐7a and bioinformatics analysis showed that circRNA_101128 was potentially involved in MAPK and P13K‐Akt pathway possibly via modulation of let‐7a. Taken together, our results indicated that some dysregulated circRNAs were potential biomarkers for the diagnosis of TB and circRNA_101128‐let‐7a interplay may play considerable role in PBMCs response to Mtb infection.

## 1 INTRODUCTION

Tuberculosis (TB) remains a common deadliest and communicable disease caused by *Mycobacterium tuberculosis *(Mtb), especially in low‐ and middle‐income countries.^1^ Early identification and proper therapy can obviously improve active TB patients' outcome.^2^ Great advances and many efforts have been made to treat and understand basal mechanisms of TB infection in recent years. However, remission rates remain suboptimal and TB diagnosis is still challenging due to the limitations in the specificity and sensitivity of the current diagnostic tests.^3^ It may be because the underlying molecular mechanism of TB remains largely elusive.

Genetic alterations are the key to revealing the pathogenesis of active TB. Circular RNAs (circRNAs), a recently discovered class of endogenous non‐coding RNAs, have been shown to be abundantly expressed in human cells and characterized by covalently closed continuous loop without free 3′ or 5′ ends, which confers them high resistance to RNase R.^4^ Therefore, this makes circRNAs to be more suitable diagnostic biomarkers than other types of RNA. Meanwhile, emerging evidence has suggested that circRNAs play crucial roles in the regulation of gene expression at both transcriptional and post‐transcriptional levels, and their tissue‐specific as well as disease‐associated expression patterns have been proposed as significant regulators for underlying pathological mechanisms and therapeutic targets for diseases.^5^ However, to our knowledge, their expression patterns and functions in active TB are still limited.

This study was designed to determine whether circRNAs in peripheral blood mononuclear cells (PBMCs) could be used as novel biomarkers for active TB diagnosis and whether aberrantly expressed circRNAs may provide new suggestion for further understanding of the potential mechanisms of TB pathogenesis and therapeutic strategy.

## 2 MATERIALS AND METHODS

### 2.1 Participants and PBMC samples preparation

Sixty‐one participants were included in the study: active TB patients (PTC group, n = 31) and healthy controls (PCR group, n = 30). Patients with active TB were recruited from Weifang Respiratory Disease Hospital in Shandong Province, China, and all patients were diagnosed on the basis of the typical TB clinical symptoms and sputum smear or culture positive. In order to have a relatively homogenous population, only patients without obvious cavity in lungs were enrolled in the study. Moreover, patients were excluded if they received any type of treatment or meanwhile suffered from other diseases like pneumonia, diabetes, cancer, acquired immune deficiency syndrome (AIDS), hepatitis B or C. Age‐ and gender‐matched healthy patients were from the Affiliated Hospital of Weifang Medical University and served as controls in the study. In addition, healthy controls with recent close contact of TB patient or history of TB were excluded from the study. Meanwhile, we carried out interferon‐γ release assay to detect whether the controls were not latently infected with Mtb. The basic demographic characteristics of all the participants in the study were summarized in Table S1.

Fasting venous blood samples were obtained at diagnosis prior to treatment initiation. After sample collection, PBMCs were freshly isolated by Ficoll gradient centrifugation. Resulting PBMC samples were aliquoted and immediately kept in liquid nitrogen until further analysis. Three PBMC specimens were choosed randomly from each group (male/female = 2/1) and were used for microarray analysis. All of the PBMC samples were applied for subsequent reverse transcription‐quantitative polymerase chain reaction (RT‐qPCR) verification.

All study protocols were approved by Weifang Medical University local ethics committee and were carried out in compliance with the Code of Ethics of the World Medical Association (Declaration of Helsinki). Written informed consent was obtained from all the participants prior to their enrolment in the study.

### 2.2 Total RNA isolation and microarray hybridization

Total RNA from PMBC samples were extracted with TRIzol^®^ reagent (Invitrogen, Waltham, MA, USA) based on the manufacturer's protocol. The purity and concentration of total RNA were assessed with a NanoDrop ND‐1000 instrument (Thermo Fisher Scientific, Inc., Waltham, MA, USA) and electrophoresis analysis. Only qualified RNA sample can be used for further microarray hybridization analysis.

Sample labelling and microarray hybridization were performed according to the Arraystar's standard instructions (Arraystar Inc., Rockville, MD, USA). Briefly, qualified total RNA was digested with RNase R (Epicentre, Inc., Madison, WI, USA) to enrich circRNAs. Then, the resulting circRNA samples were labelled using random priming method (Arraystar Super RNA Labeling Kit, Arraystar Inc.) and were purified with a RNeasy Mini Kit (Qiagen, Inc., Valencia, CA, USA). Subsequently, the qualified labelled circRNA samples with the yield ＞1.65 μg and the specific activity ＞9.0 pmol Cy3/μg cRNA were hybridized onto Arraystar Human circRNA Arrays containing 13 617 probes (V2, Arraystar Inc.) and the microarray slides were then incubated for 17 hours at 65°C in an Agilent Hybridization Oven (Agilent Technologies, Inc., Santa Clara, CA, USA). After hybridization, microarray slides were then washed, fixed and scanned utilizing an Agilent Scanner G2505C (Agilent Technologies, Inc.).

For data analysis**,** Agilent Feature Extraction software (version 11.0.1.1) was applied to analyse the acquired slide images. Then, r software package (r version 3.1.2) was used for quantile normalization of raw data and subsequent data processing. Next, low intensity filtering was carried out and the circRNAs that at least three out of six samples have flags in “P” or “M” (“All Targets Value”) were left for further analysis. CircRNAs with both false discovery rate (FDR) < 0.05 and fold‐change (FC) value >2 were considered to be differentially expressed. Moreover, scatter plot was performed to show the variation in circRNA expression and volcano plot filtering was used to identify differentially expressed circRNAs with statistical significance. Meanwhile, hierarchical clustering analysis was applied to show the dysregulated circRNAs expression profile among the samples.

### 2.3 Reverse transcription‐quantitative polymerase chain reaction

Total RNA was prepared from each PBMC sample using TRIzol^®^ reagent (Invitrogen), and then, PrimeSuperScript^TM^Ⅱ(TaKaRa,)was utilized to synthesize cDNA templates from 200 ng of total RNA. qPCR was performed in a volume of 20 μL containing 2 μL of cDNA and 0.5 μmol/L of each forward and reverse primers (primer sequences were available if required). PCR thermocycling conditions were set at 95°C for 10 minutes for pre‐denaturation, followed by 40 cycles at 95°C for 10 seconds and 60°C for 60 seconds. Housekeeping gene GAPDH was applied as an internal reference. All reactions were performed in triplicate and the $2^{-\Delta \Delta Ct}$ method was used to calculate relative gene expression levels.

### 2.4 Receiver operating characteristic analysis

Receiver operating characteristic curve (ROC) was constructed and the area under the curve (AUC) value as well as 95% confidence intervals (CI) were calculated to evaluate the predictive power of the selected circRNAs for active TB diagnosis.

### 2.5 Annotation for circRNA/miRNA interaction and KEGG pathway analysis

circRNA/miRNA interaction was predicted with Arraystar's home‐made miRNA target prediction software TargetScan (http://www.targetscan.org/) and miRanda (http://www.microrna.org/) to annotate miRNA response elements (MREs) potentially targeted by the confirmed dysregulated circRNAs based on their seed‐sequence complementarity. Function of hsa_circRNA_101128 was predicted based on the analysis of its targeted miRNA‐mRNAs by gene ontology (GO) and Kyoto Encyclopedia of Genes and Genomes (KEGG) pathway analysis.

### 2.6 Statistical analysis

Student's *t* test and chi‐squared test were performed to evaluate the statistical significance between the two groups. Correlation between circRNA and its possible targeted miRNA among individual samples was assessed. A *P* value of <0.05 was viewed as statistically significant.

## 3 RESULTS

### 3.1 Analysis of circRNA expression profiles in PBMCs

**Figure 1:**
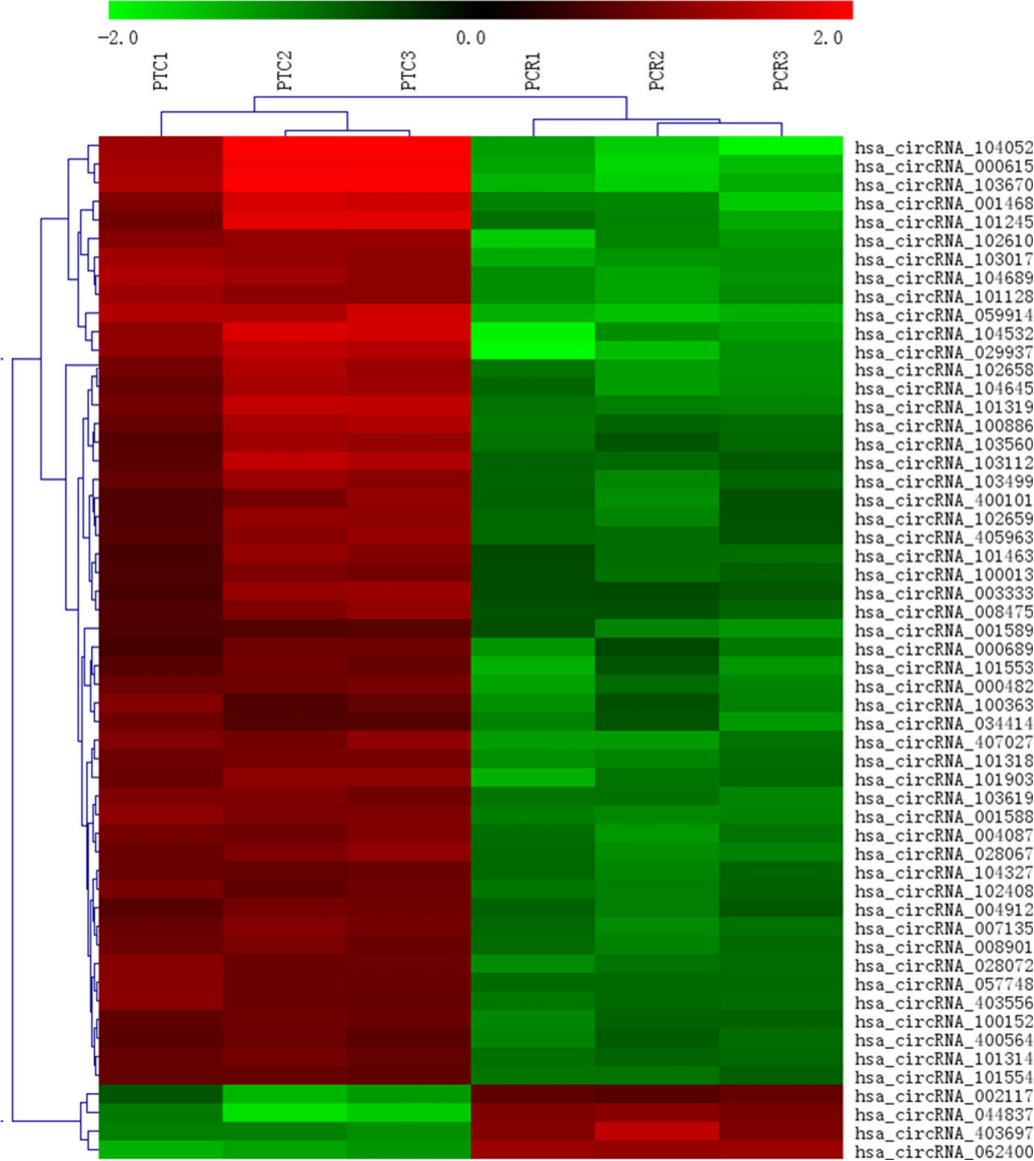
Hierarchical cluster analysis of circRNA expression in PBMCs from active TB group compared with control group. Expression of the dysregulated circRNAs with FC ＞ 3 and FDR adjusted *P* value <0.05 was depicted in the hierarchical clustering. The red and green represented an increased and a decreased expression of circRNAs, respectively. Cluster analysis indicated that circRNAs expression pattern was altered in PBMCs from active TB patients. PBMC: peripheral blood mononuclear cells; FC: fold change; FDR: false discovery rate; PTC: active TB group; PCR: control group

To identify the potential circRNAs related to human PBMCs response to Mtb infection, we characterized the circRNAs expression profiles using microarray assays. As present in FigureS1A and B, scatter plot visualization showed the normalized expression level of circRNAs and volcano plot displayed the differential expression of circRNAs. When the screening criteria were set as FC ＞ 2 and *P* < 0.05, a total of 171 circRNAs (FC ＞ 2 and *P* value <0.05 adjusted with FDR) were found to be differentially expressed; of these, 145 were up‐regulated while 26 were down‐regulated in the active TB group compared with the healthy controls. Particularly, we found that hsa_circRNA_059914 was the highest while hsa_circRNA_062400 showed the largest down‐regulation in the TB group, respectively. Furthermore, when the screening criteria were set as FC ＞ 3 and *P* < 0.05, 55 circRNAs were identified to be significantly dysregulated (Table S2), of which, 51 were increased while four were decreased in TB samples. At the same time, hierarchical cluster analysis was used to visualize the differential expression patterns of the 55 circRNAs among the samples in the present study (Figure 1). Our results demonstrate that circRNAs alterations are involved in human PBMCs response to Mtb infection.

Microarray expression data set in this study has been submitted to the Gene Expression Omnibus (GEO, http://www.ncbi.nlm.nih.gov/geo) database and the GEO accession number is GSE117563.

### 3.2 Confirmation of circRNA expression

**Figure 2:**
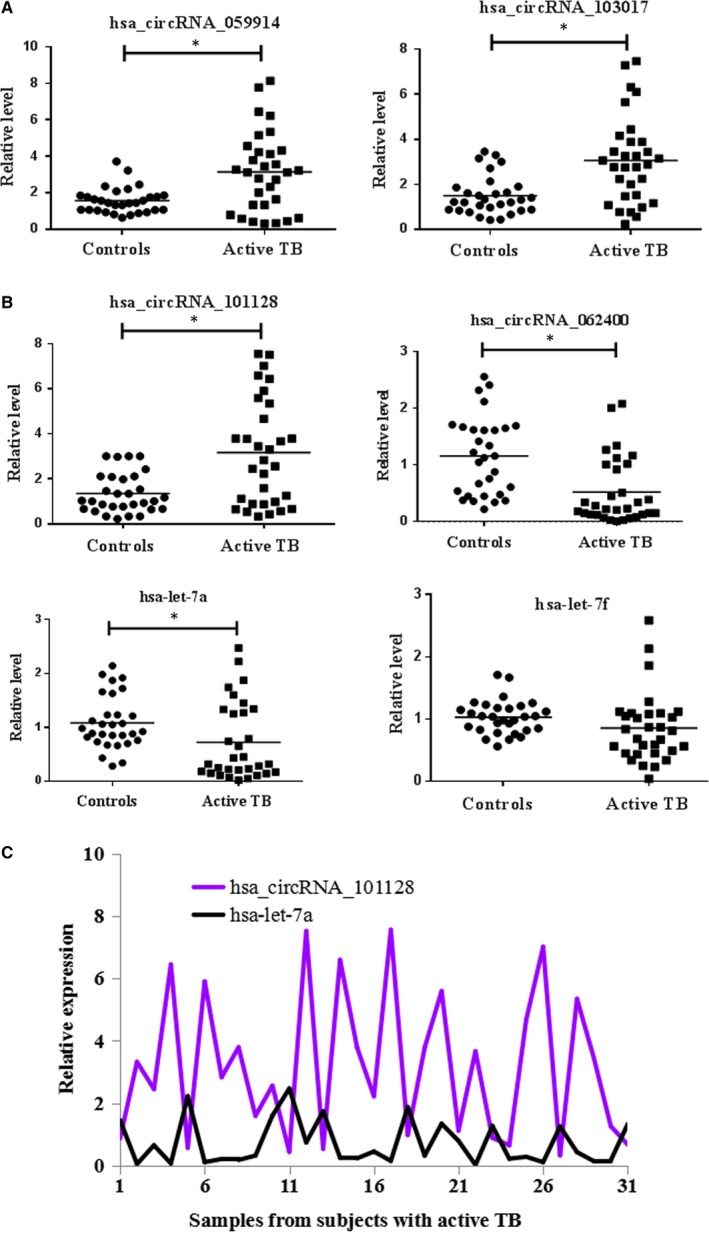
RT‐qPCR analysis of selected circRNAs and miRNAs. (A) Confirmation of the expression of candidate differentially expressed circRNAs**. **hsa_circRNA_103017, hsa_circRNA_059914 and hsa_circRNA_101128 were enhanced, while hsa_circRNA_062400 was reduced in the active TB patient group (n = 31) vs healthy patient group (n = 30). (B) Prediction of possible miRNA targets of hsa_circRNA_101128. hsa‐let‐7a and hsa‐let‐7f were decreased in the case group (n = 31) compared with the controls (n = 30). (C) Expression level of hsa_circRNA_101128 was negatively correlated with that of let‐7a (*r* = −0.56, *P* ＜ 0.05)**. **GAPDH and U6 were applied as reference genes of circRNAs and miRNAs, respectively. Data were analysed with the $2^{-\Delta \Delta C_{t}}$ method. Student's *t *test was utilized for statistical analysis. * *P* < 0.05 vs controls

To further verify the microarray results, the top three increased (hsa_circRNA_059914, hsa_circRNA_103017 and hsa_circRNA_101128) and the top one decreased circRNAs (hsa_circRNA_062400) were selected for confirmation by RT‐qPCR in a total of 61 samples from 31 active TB patients and 30 healthy individuals. As shown in Figure 2A, hsa_circRNA_059914, hsa_circRNA_103017 and hsa_circRNA_101128 were significantly higher, while hsa_circRNA_062400 was obviously lower in TB samples as compared to healthy controls (*P* < 0.05). Our findings confirmed the data of the microarray analysis.

### 3.3 Diagnostic value of the confirmed circRNAs in TB infection

**Figure 3:**
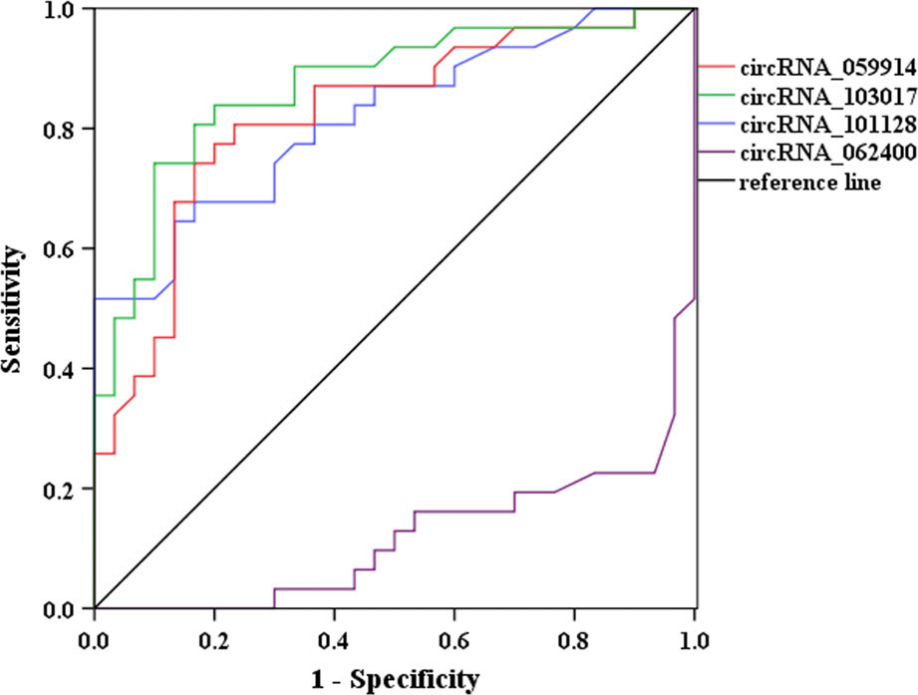
ROC analysis of differentially expressed circRNAs between patients with active TB and healthy patients.** **hsa_circRNA_103017, hsa_circRNA_059914 and hsa_circRNA_101128 showed good distinguishing efficiency with AUC values of 0.870 (95% CI, 0.779‐0.961), 0.821 (95% CI, 0.714‐0.928) and 0.817 (95% CI, 0.712‐0.922), respectively. However, hsa_circRNA_062400 exhibited poor ability to discriminate active TB patients from healthy controls. ROC, Receiver operating characteristic

Recently, many studies have suggested that circRNAs can serve as novel diagnostic markers for diseases. Therefore, ROC curve was calculated to assess whether the four confirmed circRNAs serve as diagnostic biomarkers for TB infection. As shown in Figure 3, the highest AUC was found for hsa_circRNA_103017 (AUC: 0.870, 95% CI: 0.779‐0.961, *P < *0.001), followed by hsa_circRNA_059914 (AUC: 0.821, 95% CI: 0.714‐0.928, *P < *0.001) and hsa_circRNA_101128 (AUC: 0.817, 95% CI: 0.712‐0.922, *P* < 0.001). These findings indicate that hsa_circRNA_103017, hsa_circRNA_059914 and hsa_circRNA_101128 may hold promise as potential novel biomarkers for the diagnosis of active TB. However, hsa_circRNA_062400 displayed poor ability to discriminate TB patients from healthy controls.

### 3.4 Annotation for circRNA/miRNA interaction

**Figure 4:**
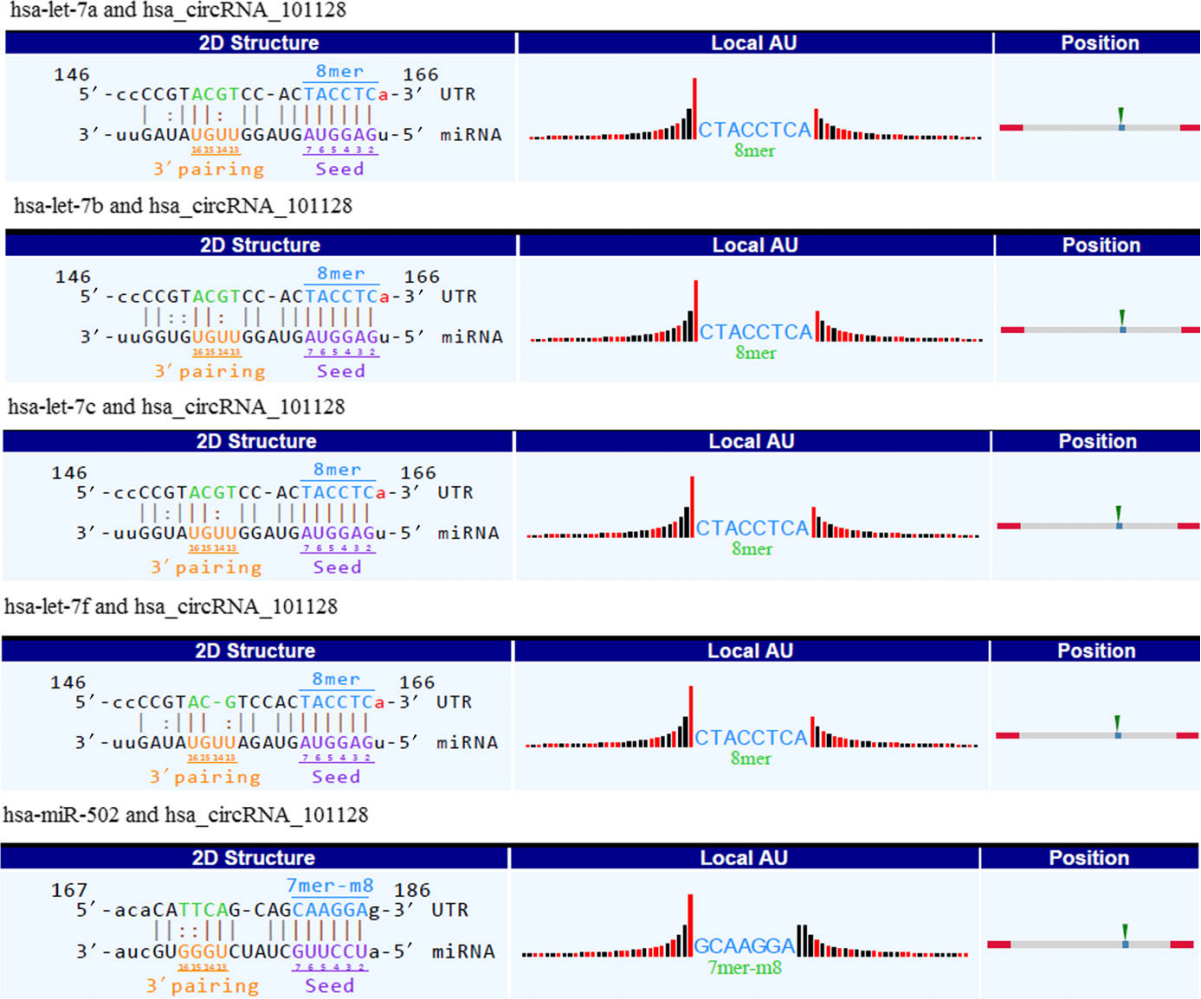
Detailed presentation of interactions between hsa_circRNA_101128 and its five potential miRNA targets. The 2D structure displayed the MRE sequence, pairing of miRNA target nucleotides and seed type. The “local AU” showed upstream and downstream of the seed sequence, black and red bars represented G/C and low accessibility as well as A/U and high accessibility of the seed, respectively, and bar height stands for the extent of accessibility. The position column showed the most likely relative MRE position on the linearized circRNA sequence. MRE, microRNA response elements

Recent studies have demonstrated that circRNAs play an important role in acting as an endogenous “sponge” to sequester miRNA‐mediated regulation of mRNA expression,^6^, ^7^ which suggests that circRNAs have crucial functions in the regulation of miRNA expression. To investigate circRNAs possible functions, we analysed potential miRNAs binding with circRNAs using Arraystar's home‐made miRNA target prediction software. In this study, we focussed on four confirmed circRNAs (hsa_circRNA_103017, hsa_circRNA_059914, hsa_circRNA_101128 and hsa_circRNA_062400) to predict their MREs. Five MREs with good mirSVR scores for each circRNA were present in Table 1; of these miRNAs, it is worth noting that certain miRNAs have been reported to be dysregulated in TB, such as let‐7a and let‐7f,^8^, ^9^, ^10^ which were decreased upon Mtb infection. More importantly, these above‐mentioned miRNAs were possible targets of hsa_circRNA_101128, which was up‐regulated in this study. The detailed potential interplay between hsa_circRNA_101128 and its target miRNAs including let‐7a and let‐7f was shown based on sequence‐pairing miRNA target prediction (Figure 4). To further investigate whether there was a relationship between hsa_circRNA_101128 and let‐7a as well as let‐7f in PBMCs, expression of both miRNAs was detected using RT‐qPCR. We found that let‐7a was reduced (Figure 2B), while let‐7f did not show any obvious difference in the TB group vs the controls. Furthermore, correlation analysis exhibited that, there was negative correlation between hsa_circRNA_101128 expression and let‐7a level in TB samples (*r* = −0.56, *P* ＜ 0.05) (Figure 2C), while there was no correlation in control samples (data not shown). The data suggested that hsa_circRNA_101128 was potentially involved in the pathogenesis of active TB via negative regulation of let‐7a.

**Table 1:** Dysregulated circRNAs verified by RT‐qPCR and MREs

CircRNA	MRE1	MRE2	MRE3	MRE4	MRE5
hsa_circRNA_059914	hsa‐miR‐377‐5p	hsa‐miR‐6086	hsa‐miR‐4756‐3p	hsa‐miR‐103b	hsa‐miR‐767‐3p
hsa_circRNA_103017	hsa‐ miR‐1‐3p	hsa‐miR‐449c‐5p	hsa‐miR‐206	hsa‐miR‐34b‐5p	hsa‐miR‐122‐5p
hsa_circRNA_101128	hsa‐let‐7c‐5p	hsa‐ let‐7b‐5p	hsa‐ let‐7a‐5p	hsa‐ let‐7f‐5p	hsa‐miR‐502‐5p
hsa_circRNA_062400	hsa‐miR‐7843‐5p	hsa‐miR‐4656	hsa‐miR‐6768‐3p	hsa‐miR‐4436b‐3p	hsa‐miR‐6511a‐5p

MRE, microRNA response element.

Let‐7 can regulate the immune reponse to Mtb infection and control bacterial burden by modulation of NF‐κB pathway.^10^ Considering the negative correlation between hsa_circRNA_101128 and let‐7, this inspired us to focus on the biological significance of has_circRNA_101128 in active TB. To further uncover the role of hsa_circRNA_101128, GO and KEGG pathway enrichment analysis was conducted to elucidate its possible biological functions based on analysis of let‐7a‐target mRNAs. GO results showed that the main biological processes (BP) were pre‐miRNA processing, protein phosphorylation, cellular response to amino acid stimulus, miRNA loading onto RNA‐induced silencing complex (RISC) and production of miRNAs involved in gene silencing by miRNA; the main cellular components were endoplasmic reticulum lumen, RISC complex and micro‐ribonucleoprotein complex, nucleoplasm and nucleus; and the main molecular functions were protein kinase activity, protein binding, metal ion binding, extracellular matrix structural constituent as well as miRNA binding. KEGG data suggested that the pathways were predominantly involved in MAPK signalling pathway, P13K‐Akt signalling pathway, FoxO signalling pathway, protein digestion and absorption (Table S3).

## 4 DISCUSSION

As TB is one of the leading causes of death caused by a single pathogen worldwide, it is imperative to identify novel diagnostic biomarkers and therapeutic targets to improve patient's survival. Recent evidence has indicated that circRNA plays important roles in various pathological processes and its dysregulation has also been involved in the occurrence and progression of many diseases, such as viral infection.^11^, ^12^ Due to its important function, relationship between circRNA and TB infection has started to be gained attention in the past 2 years and it was found that circRNAs dysregulation was involved in Mtb infection. For example, hsa_circ_0005836 was reported to act as a new possible biomarker for active TB disease^13^; circRNA_00074, circRNA_09585, circRNA_14623, circRNA_005538, circRNA_09993 and circRNA_13478 were found to be differentially expressed in leukocytes from active TB patients.^14^ These findings imply that circRNAs may play important roles in active TB and have the great potential to serve as a novel type of biomarkers for TB diagnosis. Despite some efforts devoted to uncovering circRNAs profiling during the last 2 years, in reality, the knowledge of circRNAs in active TB still remains in its infancy stage.

In this study, we conducted a comprehensive microarray analysis of global changes in the expression pattern of circRNAs in PBMCs from active TB patients. We found that 171 circRNAs were dysregulated in TB samples**,** which implicated that these aberrantly expressed circRNAs were potentially associated with TB infection. Of them, 145 circRNAs were elevated while only 26 circRNAs were reduced in TB samples compared with the controls, which were in line with the report that the circRNA's magnitude in PBMCs from active TB patients was significantly increased.^15^ Moreover, our results showed that the expression pattern of circRNAs in PBMCs is similar to that in plasma,^16^ where the most dysregulated circRNAs were enhanced upon Mtb infection. This indicates that circRNAs are possibly turned on to promote the pathogenesis and progression of active TB, which might facilitate our understanding of circRNA functions in active TB. However, functions of these dysregulated circRNAs are largely unknown.

In this study, four dysregulated circRNAs were selected for further confirmation using RT‐qPCR. hsa_circRNA_059914, the top first increased circRNA in TB samples**,** is derived from the *AHCY *gene and its encoded S‐adenosylhomocysteine hydrolase is associated with many diseases such as hepatocellular carcinoma.^17^ hsa_circRNA_103017, the top second elevated circRNA, is encoded by the *ASXL1* gene, which encodes a well‐known tumour suppressor protein and its mutation plays an important role in myeloid neoplasms.^18^ hsa_circRNA_101128, the top third up‐regulated circRNA, aligns with *CORO1C *gene, which encodes a cytoskeleton‐associated protein related to tumour metastasis.^19^ hsa_circRNA_062400, the top first decreased circRNA, is derived from the *CRKL* gene, and its encoded protein is involved in tumour metastasis.^20^ However, roles of these circRNA‐related protein‐coding genes in active TB remain little known. Elucidating underlying molecular function of the coding genes that links circRNA and TB pathogenesis is particularly important for developing predictive biomarkers and therapeutic targets for improving TB patients treatment outcome.

To further address the feasibility of these verified circRNAs as potential biomarkers for active TB diagnosis, ROC curve was applied to evaluate their diagnostic value. We found that hsa_circRNA_059914, hsa_circRNA_103017 and hsa_circRNA_101128 exhibited significant distinguishing efficiency to discriminate between TB cases and healthy individuals. However, the three dysregulated circRNAs were not observed in the previous findings mentioned above.^13^, ^15^, ^21^ This discrepancy may be due to samples from different stage of active TB and small sample size in the confirmation phase. However, there is no doubt that the data suggest the clinical potential for the different circRNAs profile to serve as appropriate biomarkers for diagnosis of active TB with more validation based on larger cohorts.

We further focussed on hsa_circRNA_101128, which was predicted to target let‐7a and let‐7f based on bioinformatics analysis, and interestingly, these miRNAs have been shown to be associated with active TB.^8^, ^9^, ^10^ CircRNAs have been reported to be predominantly in the cytoplasm and can action as miRNA sponges to play essential regulatory roles during the development and progression of many diseases.^22^, ^23^ Considering this, we suppose that whether let‐7a or let‐7f has correlation with hsa_circRNA_101128. The results in the current study displayed that hsa_circRNA_101128 expression was negatively correlated with let‐7a level, which indicated that hsa_circRNA_101128 might be involved in the pathogenesis of active TB by negative modulation of let‐7a. Moreover, we further investigated the role of hsa_circRNA_101128 based on bioinformatics analysis. Indeed, KEGG analysis showed that the top signalling pathways MAPK and P13K‐Akt were enriched, which have been reported to be associated with active TB.^24^, ^25^ This may provide useful information for further exploring potential roles of circRNA in the development and progression of active TB. However, more samples are further required for validation and functional studies are needed to investigate the roles of hsa_circRNA_101128 in active TB.

This is the first study to show that hsa_circ_103017, hsa_circ_059914 and hsa_circ_101128 in PBMCs are potential biomarkers for the diagnosis of active TB and suggest that hsa_circRNA_101128 may play a considerable role in the pathogenesis of active TB possibly through regulation of let‐7a. Taken together, these findings of the study may help with future studies to identify new diagnostic markers, therapeutic targets for active TB and understand the pathogenesis of the disease. However, these data are from a small sample of only 31 patients, and further experimental explorations of larger clinical sample cohorts are needed to evaluate and validate the results. Furthermore, the functional implications of the differentially expressed circRNAs await systematic investigations.

## CONFLICTS OF INTEREST

All the authors declare that there are no conflicts of interest relevant to this article.
