# Supplementary material for: Signature of circular RNAs in peripheral blood mononuclear cells from patients with active tuberculosis
Source: J Cell Mol Med. 2018 Dec 18;23(3):1917–25. doi: 10.1111/jcmm.14093 (PMC6378186; doi:10.1111/jcmm.14093)
Supplement: Supplementary file 1 [file JCMM-23-1917-s001.doc]

**Supporting information**


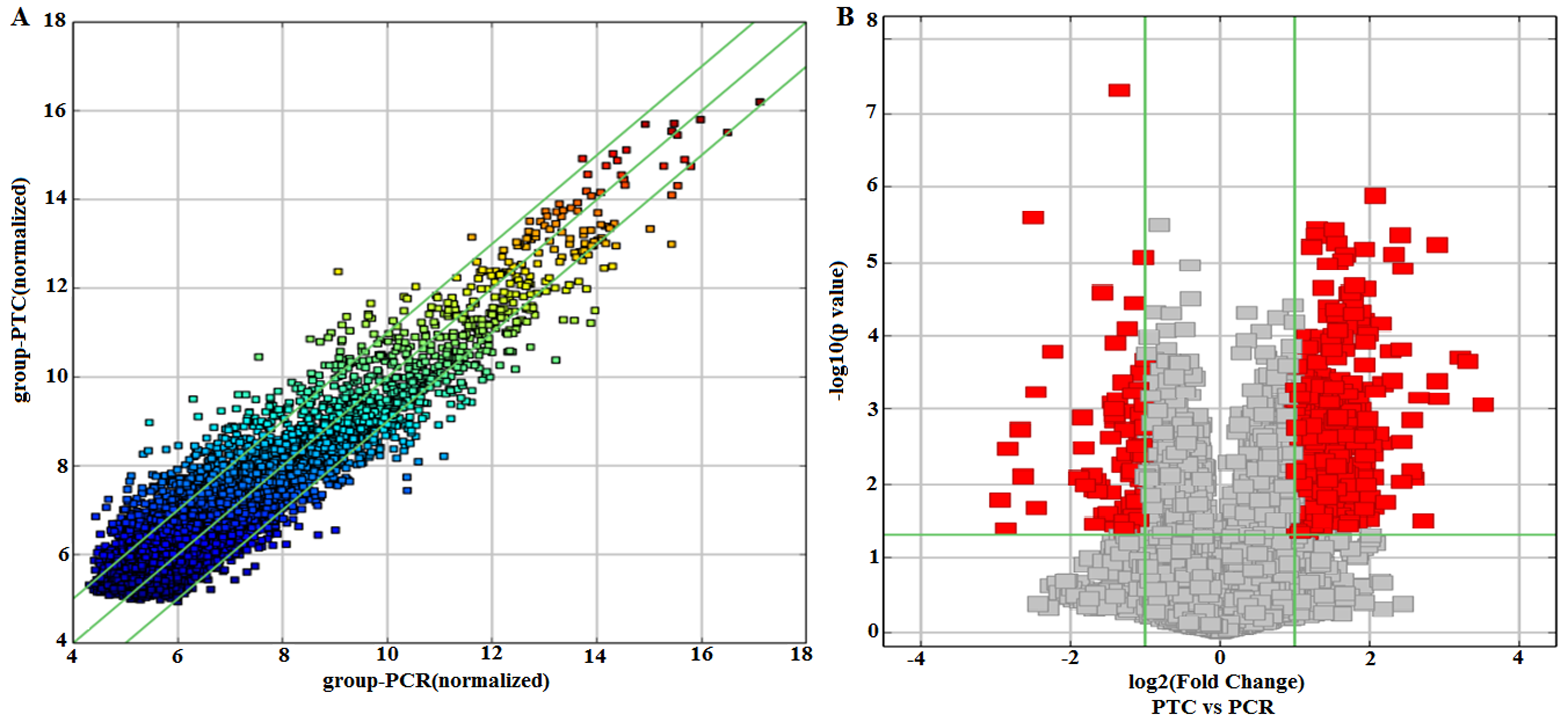
[**Fig**](https://www.ncbi.nlm.nih.gov/pmc/articles/PMC5835825/figure/fig01/)**ure S1 CircRNAs profile expression in PBMCs from active TB patients versus controls**

A. Scatter plot analysis. The values spotted in the X and Y axes indicate the normalized signals. The circRNAs above the upper green line and below the lower green line are those with expression FC more than 2 between the two groups. B. Volcano plot analysis. The vertical lines represent 2 FC upregulation or downregulation, and the horizontal line represents a FDR of 0.05. The red points in the plot indicate the deregulated circRNAs with FC >2 and FDR adjusted *P* value <0.05. PBMC: peripheral blood mononuclear cells; FC: fold change; FDR: false discovery rate; PTC: active TB group; PCR: control group.

**Table S1 Clinical characters of the study par­ticipants**

| Characters | PTC group (n=31) | PCR group (n=30) |
| --- | --- | --- |
| Mean age,years(range) | 38.9±15.7 (20-56) | 36.6±14.5 (22-53) |
| Male/female | 17/14 | 16/14 |
| TST test | NA | — |
| IGRA | NA | — |
| COPD  Asthma  Pneumonia  Diabetes | —  —  —  — | —  —  —  — |
| Cancer | — | — |
| Hypertension  HIV | —  — | —  — |
| HBV | — | — |
| HCV | — | — |

PTC group, active TB patients group; PCR group, healthy subjects group; TST, tuberculin skin test; IGRA, interferon-Gamma release assays; COPD, chronic obstructive pulmonary disease; NA, no applicable; —, negative. There was no significant difference in age (*P* >0.05) or gender (*P* >0.05) between the TB case group and the healthy controls.

**Table S2** Differentially expressed circRNAs in the active TB group compared with the controls

| **circRNAs** | **GeneSymbol** | **circRNA type** | **chrom** | **FC** | **FDR** |
| --- | --- | --- | --- | --- | --- |
| hsa_circRNA_059914 | AHCY | exonic | chr20 | 7.488 | 0.004 |
| hsa_circRNA_103017 | ASXL1 | exonic | chr20 | 5.303 | 0.004 |
| hsa_circRNA_101128 | CORO1C | exonic | chr12 | 5.013 | 0.004 |
| hsa_circRNA_001588 | HIST1H4E | sense overlapping | chr6 | 4.208 | 0.004 |
| hsa_circRNA_103619 | RELL1 | exonic | chr4 | 3.807 | 0.004 |
| hsa_circRNA_101314 | HNRNPC | exonic | chr14 | 3.186 | 0.004 |
| hsa_circRNA_104689 | ASAP1 | exonic | chr8 | 5.435 | 0.005 |
| hsa_circRNA_101554 | SNX1 | exonic | chr15 | 3.122 | 0.005 |
| hsa_circRNA_101318 | RBM23 | exonic | chr14 | 3.848 | 0.008 |
| hsa_circRNA_008901 | PTPN12 | exonic | chr7 | 3.476 | 0.008 |
| hsa_circRNA_057748 | CFLAR | exonic | chr2 | 3.360 | 0.009 |
| hsa_circRNA_104327 | FAM126A | exonic | chr7 | 3.304 | 0.009 |
| hsa_circRNA_102408 | THOP1 | exonic | chr19 | 3.422 | 0.010 |
| hsa_circRNA_028072 | CORO1C | exonic | chr12 | 3.690 | 0.011 |
| hsa_circRNA_403556 | LINC00340 | exonic | chr6 | 3.445 | 0.011 |
| hsa_circRNA_004087 | CDYL2 | exonic | chr16 | 3.806 | 0.012 |
| hsa_circRNA_007135 | CCZ1B | exonic | chr7 | 3.653 | 0.012 |
| hsa_circRNA_407027 | RHEB | exonic | chr7 | 4.437 | 0.013 |
| hsa_circRNA_100152 | ZBTB8OS | exonic | chr1 | 3.250 | 0.014 |
| hsa_circRNA_400564 | REEP3 | exonic | chr10 | 3.118 | 0.016 |
| hsa_circRNA_028067 | CORO1C | exonic | chr12 | 3.941 | 0.016 |
| hsa_circRNA_000482 | SLC45A4 | exonic | chr8 | 3.841 | 0.017 |
| hsa_circRNA_102610 | MBOAT2 | exonic | chr2 | 5.361 | 0.019 |
| hsa_circRNA_102658 | MEMO1 | exonic | chr2 | 4.856 | 0.019 |
| hsa_circRNA_403697 | MCM9 | exonic | chr6 | 4.716 | 0.019 |
| hsa_circRNA_004912 | PHF7 | exonic | chr3 | 3.160 | 0.019 |
| hsa_circRNA_000615 | ZNF609 | sense overlapping | chr15 | 9.264 | 0.021 |
| hsa_circRNA_103670 | CNOT6L | exonic | chr4 | 9.922 | 0.022 |
| hsa_circRNA_103499 | RSRC1 | exonic | chr3 | 3.814 | 0.022 |
| hsa_circRNA_029937 | N4BP2L2 | exonic | chr13 | 7.480 | 0.027 |
| hsa_circRNA_101319 | RBM23 | exonic | chr14 | 4.929 | 0.027 |
| hsa_circRNA_100013 | GNB1 | exonic | chr1 | 3.031 | 0.027 |
| hsa_circRNA_104645 | STAU2 | exonic | chr8 | 4.517 | 0.028 |
| hsa_circRNA_405963 | UGGT1 | intronic | chr2 | 3.396 | 0.028 |
| hsa_circRNA_100886 | RAB6A | exonic | chr11 | 4.116 | 0.031 |
| hsa_circRNA_008475 | ARPC2 | exonic | chr2 | 3.112 | 0.031 |
| hsa_circRNA_101903 | BANP | exonic | chr16 | 4.204 | 0.032 |
| hsa_circRNA_034414 | RASGRP1 | exonic | chr15 | 3.271 | 0.032 |
| hsa_circRNA_001589 | HIST1H1D | sense overlapping | chr6 | 3.085 | 0.032 |
| hsa_circRNA_104532 | RBM33 | exonic | chr7 | 7.566 | 0.034 |
| hsa_circRNA_001468 | MED13L | sense overlapping | chr12 | 6.308 | 0.034 |
| hsa_circRNA_103560 | UBXN7 | exonic | chr3 | 3.594 | 0.034 |
| hsa_circRNA_100363 | CCT3 | exonic | chr1 | 3.346 | 0.037 |
| hsa_circRNA_102659 | MEMO1 | exonic | chr2 | 3.499 | 0.038 |
| hsa_circRNA_101463 | UBE3A | exonic | chr15 | 3.242 | 0.040 |
| hsa_circRNA_101553 | SNX1 | exonic | chr15 | 3.679 | 0.041 |
| hsa_circRNA_400101 | RPL7A | intronic | chr9 | 3.372 | 0.042 |
| hsa_circRNA_101245 | N4BP2L2 | exonic | chr13 | 5.929 | 0.045 |
| hsa_circRNA_103112 | USP25 | exonic | chr21 | 3.920 | 0.045 |
| hsa_circRNA_000689 | MBOAT2 | intronic | chr2 | 3.188 | 0.045 |
| hsa_circRNA_003333 | MCTP2 | exonic | chr15 | 3.080 | 0.046 |
| *hsa_circRNA_062400* | *CRKL* | *exonic* | *chr22* | *5.659* | *0.004* |
| *hsa_circRNA_044837* | *YPEL2* | *exonic* | *chr17* | *5.515* | *0.032* |
| *hsa_circRNA_104052* | *CDYL* | *exonic* | *chr6* | *11.447* | *0.038* |
| *hsa_circRNA_002117* | *PC* | *intronic* | *chr11* | *3.580* | *0.045* |
|  |  |  |  |  |  |

Fifty-five deregulated circRNAs with FC＞3 and FDR < 0.05 were identified between the two groups. Among these differentially expressed circRNAs, 51 circRNAs were upregulated and only 4 circRNAs (the last 4 italic ones in the table) were downregulated in the active TB group *versu*s the controls.

**Table S3** KEGG enrichment analysis for hsa_circRNA_101128

| Pathway name | Gene count | *P*-value | Benjamini |
| --- | --- | --- | --- |
| MAPK signaling pathway | 38 | 4.8E-7 | 1.2E-4 |
| P13K-Akt signaling pathway | 42 | 2.1E-5 | 1.8E-3 |
| [FoxO signaling pathway](https://david.ncifcrf.gov/kegg.jsp?path=hsa04068$FoxO signaling pathway&termId=550028751&source=kegg) | 22 | 4.9E-5 | 2.5E-3 |
| [Protein digestion and absorption](https://david.ncifcrf.gov/kegg.jsp?path=hsa04974$Protein digestion and absorption&termId=550028855&source=kegg) | 17 | 6.4E-5 | 2.7E-3 |
| Pathways in cancer | 45 | 4.5E-5 | 2.9E-3 |
| P53 signaling pathway | 14 | 1.6E-4 | 4.4E-3 |
| [Focal adhesion](https://david.ncifcrf.gov/kegg.jsp?path=hsa04510$Focal adhesion&termId=550028783&source=kegg) | 27 | 2.7E-4 | 6.9E-3 |
| ECM-receptor interaction | 15 | 6.7E-4 | 1.2E-2 |
| Wnt signaling pathway | 19 | 1.6E-3 | 2.4E-2 |
| [TGF-beta signaling pathway](https://david.ncifcrf.gov/kegg.jsp?path=hsa04350$TGF-beta signaling pathway&termId=550028778&source=kegg) | 13 | 4.4E-3 | 4.8E-2 |
